# Impact of COVID-19 on non-communicable disease management services at selected government health centers in Addis Ababa, Ethiopia

**DOI:** 10.4314/ahs.v22i3.57

**Published:** 2022-09

**Authors:** Abiyu Mekonnen, Zelalem Destaw, Dejene Derseh, Eshetu Gadissa, Solomon Ali

**Affiliations:** 1 Department of Medical Laboratory Sciences, Menelik-II Medical and Health Sciences College, Kotebe Metropolitan University, Addis Ababa, Ethiopia; 2 Ethiopian Public Health Institute, Addis Ababa, Ethiopia; 3 Department of Microbiology, Saint Paul's Hospital-Millennium Medical College, Addis Ababa, Ethiopia

**Keywords:** COVID-19, non-communicable diseases, Ethiopia

## Abstract

**Background:**

The global pandemic of COVID-19 forced the world to divert resources and asked the public to shelter-in-place, so the diagnosis surveillance system and management of non-communicable diseases has become more challenging.

**Objective:**

To identify the impact of COVID-19 on non-communicable diseases management services at government health centers in Addis Ababa, Ethiopia.

**Methods:**

Health facility based cross-sectional study was conducted from August to September, 2020. A total of 30 health centers were included in this study. Bivariate and multiple logistic regression models were used to assess association between the outcome and independent variables

**Results:**

The majority, 24 (80%), of the study participants perceived that the COVID-19 pandemic severely disrupted the non-communicable disease management services. There was a statistically significant association between a decrease in outpatient volume at non communicable disease (NCD) management services (25 (83.3%), P-value: 0.006), closure of population level screening programs of NCDs (22 (73.3%), P-value: 0.007), and closure of disease specific NCD clinics and the occurrence of the COVID-19 pandemic (23 (76.7%), P-value: 0.013).

**Conclusion:**

The most critical health-care services for non-communicable diseases management were severely disrupted by the COVID-19 pandemic. Therefore, during public health emergencies, policymakers should ensure continuation of critical clinical services and inform the public about proper service utilization.

## Introduction

Coronavirus disease 2019 (COVID-19) caused by the severe acute respiratory syndrome coronavirus 2 (SARS-CoV-2) has been declared as a public health emergency of international concern by the World Health Organization (WHO) 1. Since then, COVID-19 has spread outside China to all continents causing death and economic disruption^2^.

The existing link between non-communicable diseases (NCDs), communicable diseases and health emergencies is exposed by the COVID-19 pandemic and emphasize the need to stop addressing health issues in siloes 3. Non-communicable diseases and communicable diseases like COVID-19 reinforce one another and disproportionally impacting the poorest segment of the society and the most vulnerable people around the globe 3.

In sub-Saharan Africa the magnitude of NCDs is increased over the past thirty years. The disability adjusted life years (DALYs) attributed to NCDs rose by 67% from 1990 to 2017 4; The non-communicable diseases responsible for the majority of deaths in African region were Cardiovascular disease and cancer 5, but the magnitude of diabetes was also found to be increased in the western, eastern and central Africa, while it was more prevalent in Southern Africa 6. Studies also showed that non communicable diseases such as hypertension, chronic obstructive pulmonary disease, cardiovascular disease or diabetes are each associated with increased risk of either severe disease or death due to COVID-19 7–9.

The Ministry of Health of Ethiopia has reported the first case of severe acute respiratory syndrome coronavirus 2 (SARS-CoV-2), in Addis Ababa on March 13, 2020 10; then, administrative measures including closure of schools and implementation of infection prevention measures by the public were instructed. However, the number of COVID-19 cases was increasing and a total of 132,000 cases and 2,057 deaths were reported up to January 21, 2021 11.

COVID-19 causes death, economic distraction and disruption to health services globally. Financial and human resources of the health system were mobilized to the management of COVID-19 pandemic. So, the health system responsible to manage non-communicable diseases could be interrupted. Hence, this study was intended to identify evidence about the impact of the pandemic on the management of non-communicable diseases at selected government health facilities in Addis Ababa, Ethiopia.

## Methods

### Study setting and design

The study was conducted in Addis Ababa, the capital city of Ethiopia. Addis Ababa is administratively divided into 10 sub-cities and 117 districts/Woredas. The Addis Ababa Health Bureau encompasses ninety-eight government health centers under its administration. Health facility based cross-sectional study was conducted from August to September, 2020.

### Sample size and sampling technique

The sample size for this study was determined based on recommendations from a previous study 12. Thirty or about thirty percent of the government health centers available under the administration of the Addis Ababa Health Bureau were covered. The selection of the health facilities included in the study was done by simple random sampling by using the sampling frame.

### Data collection and Statistical analysis

Data were collected by trained Nurses, Health Officers or Health Information Management System (HMIS) professionals accordingly. Nurses and Health Officers were involved in face-to-face interview, while HMIS professionals extract data from HMIS registry. A standard questionnaire designed was used to extract data from HMIS registry and face-to-face interview were conducted with the respective head of the health facility called medical director or their delegates during the data collection period. The non-communicable diseases: hypertension, diabetes mellitus and Asthma were included in this study based on the high prevalence data and availability of reasonably complete information documented in the health facilities than the other non-communicable diseases.

Face-to-face interview were conducted to obtain perceived impact and disruption of the non-communicable disease management services due to the COVID-19 pandemic. Whereas data related to the burden of non-communicable diseases in the years 2018, 2019 and 2020 and in the respective months from March 15 to July 30 were extracted from the Health Information Management System (HMIS) departments of the respective health facilities and the District Health Information Software 2 (DHIS2). DHIS2 is a free and open source health management data platform for health data collection, validation, analysis, and presentation of individual and aggregated data 13.

Descriptive statistics for predictor and outcome variables have been employed. Bivariate and multivariate logistic regression models have been used to assess the level of disruption of the health facilities non communicable diseases management services due to the COVID-19 pandemic. Furthermore, the trend of the most prevalent non communicable diseases before and after the onset of the COVID-19 pandemic was compared using figures. Data were entered into Microsoft excel and exported to IBM SPSS version-23 for statistical analysis. P-value <0.05 was considered statistically significant. Multiple regression model was fitted and Adjusted Odds Ratio (AOR) were reported with their 95% confidence interval (95% CI) in order to evaluate the level of associations between the dependent variable and the covariates.

### Ethical consideration

The study was ethically approved by the Addis Ababa Health Bureau-Public Health and Emergency Management-Ethical Review Committee (A.A.H.B.P.H.E.MERC), Reference number A/A/H/B/453/227, dated 29/07/2020. Based on the ethical clearance, permission was obtained from the respective health institutions before data collection. Each participant of the study was informed about the objectives of the study, and provided their written consent before included in the study; furthermore, no personal identifier was used during data analysis.

## Results

### Respondents' demographic characteristics

In the 30 health centers included in the study, medical directors or their delegates who participated in the study were BSc nurses or public health officers in their field of specialty. Most of the respondents were male, 20 (68.7%), with a mean age of 33 years (SD: + 4.4). The mean service year of the study participants were 4.2 years with a minimum of 2 years and a maximum of 12 years (Table-1).

**Table 1:** Socio-demographic characteristics of study participants, Addis Ababa

	Number	Percent
**Gender**		
Male	20	66.7
Female	10	31.3
**Age group**		
≤30	8	26.7
31–35	15	50.0
36–40	6	20.0
≥40	1	3.3
**Respondents service year in the health facility**		
2–5 Years	20	66.7
6–10 years	8	26.7
>10 years	2	6.7
**Respondents field of specialty**		
Public Health Officer	21	70.0
BSc Nurse	9	30.0

The majority, 24 (80%) of the study participants perceived that the COVID-19 pandemic severely disrupted the non-communicable disease (NCD) management services at the study health centers. At the same time, 25 (83.3%) described there was a decrease in the volume of outpatients for non-communicable disease management service at the health facilities. On the other hand, only about 33% stated that there were unavailability of essential medicines and diagnostics at NCD management clinics (Table-2).

**Table 2:** Perceived impact of the COVID-19 pandemic on non-communicable diseases (NCD) management services at government health centers, Addis Ababa

Variable	Number (%)
Perceived disruption rate of NCD management services due to the COVID-19 pandemic	
Severe	24 (80.0)
Moderate	6 (20.0)
Is there a decrease in outpatient volume at NCD services?	
Yes	25(83.3)
No	5 (16.7
Is there insufficient staff to provide NCD as deployed to COVID-19?	
Yes	16 (53.3)
No	14 (46.7)
Is there closure of population level screening programs of NCD?	
Yes	22 (73.3)
No	8 (26.7)
Is there transportation issue hindering NCD services?	
Yes	18 (60.0)
No	12 (40.0)
Is there closure of disease specific NCD clinics?	
Yes	23 (76.7)
No	7 (23.3)
Is there closure of NCD services as per government directions?	
Yes	15 (50.0)
No	15 (50.0)
Is there unavailability of essential medicines and Diagnostics at NCD services?	
Yes	10 (33.3)
No	20 (66.7)

There was a statistically significant association between a decrease in outpatient volume at non communicable disease management services, closure of population level screening programs of NCDs, and closure of disease specific NCD clinics and the occurrence of the COVID-19 pandemic [Crude Odds Ratio (COR) 46.0, 35.0, and 14.0, and 95% Confidence Interval (CI) of (3.33, 634.88), (2.98, 411.46) and (1.74, 112.55)], respectively (Table-3).

**Table 3:** Disruption of the Non communicable disease management services due to the COVID-19 pandemic at selected government health facilities, Addis Ababa

Variable	Disruption level		COR (95% CI)	P-Value	AOR (95%CI)	P-Value
	Severe No. (%)	Moderate No. (%)				
Is there a decrease in outpatient volume at NCD services?						
Yes	23 (76.7)	2 (6.7)	46.0 (3.33, 634.88)	0.004	48.0 (3.09, 750.03)	0.006
No	1 (3.3)	4 (13.3)	R			
Is there insufficient staff to provide NCD as deployed to COVID-19?						
Yes	14 (46.7)	2 (6.7)	0.357 (0.05,2.34)	0.283	0.32 (0.03, 4.11)	0.388
No	10 (33.3)	4 (13.3)	R			
Is there closure of population level screening programs of NCD?						
Yes	21 (70.0)	1(3.3)	35 (2.98, 411.46)	0.005	42.2 (2.78, 642.25)	0.007
No	3 (10.0)	5 (16.7)	R			
Is there transportation issue hindering NCD services?						
Yes	16 (53.3)	2 (6.7)	0.25 (0.04, 1.67)	0.152	0.18 (0.02, 2.46)	0.11
No	8 (26.7)	4 (13.3)	R			
Is there closure of disease specific NCD clinics?						
Yes	21 (70.0)	2 (6.7)	14 (1.74, 112.55)	0.013	14.7 (1.75, 123.89)	0.013
No	3 (10.0)	4 (13.3)	R			
Is there closure of NCD services as per government directions?						
Yes	12 (40.0)	3 (10.0)	1.00 (0.17, 5.99)	0.999	1.32 (0.16, 10.91	0.799
No	12 (40.0)	3 (10.0)	R			
Is there unavailability of essential medicines and Diagnostics at NCD services?						
Yes	8 (26.7)	2 (6.7)	0.99 (0.15, 6.67)	0.988	1.23 (0.13, 11.39)	0.858
No	16 (53.3)	4 (13.3)	R			

**Figure 2:**
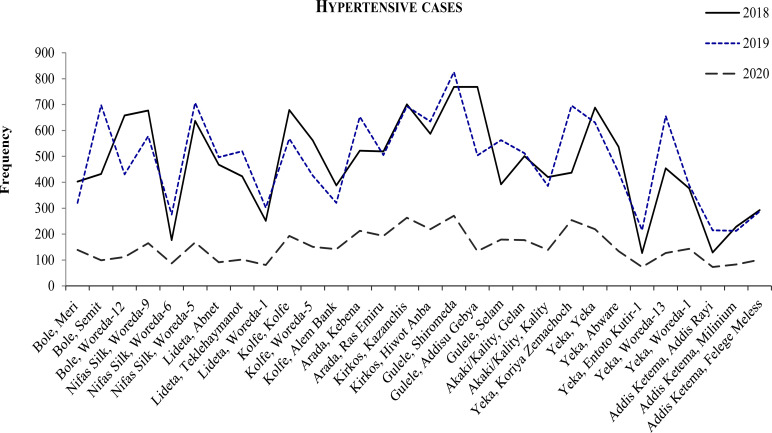
Trend of hypertensive cases in the months March-July in the respective years at selected health centers in Addis Ababa.

**Figure 3:**
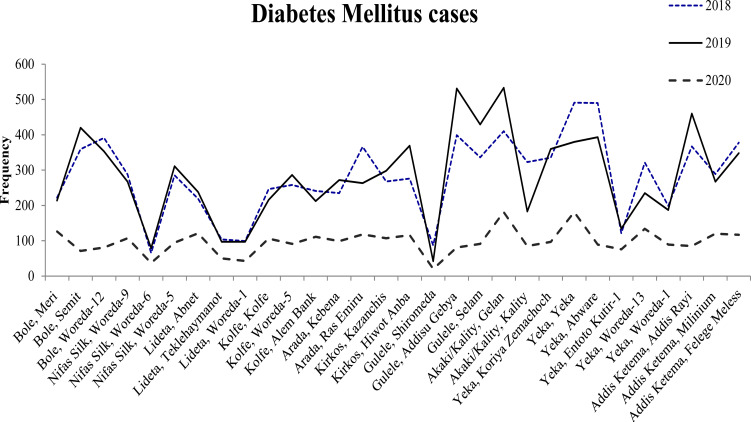
Trend of Diabetes Mellitus cases in the months March-July in the respective years at selected health centers in Addis Ababa

**Figure 4:**
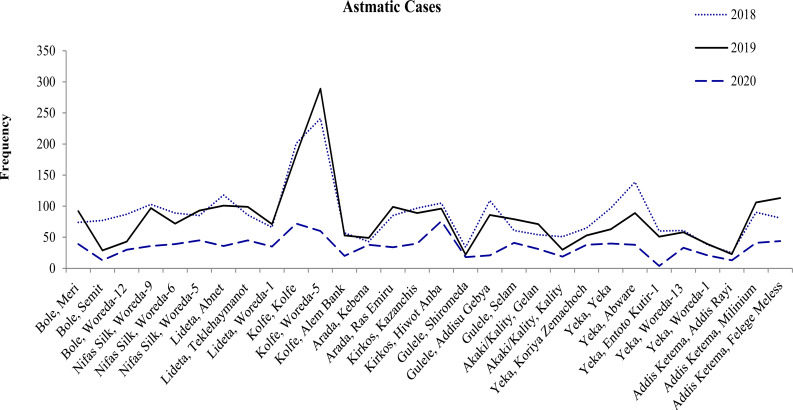
Trend of Asthmatic cases in the months March-July in the respective years at selected health centers in Addis Ababa

The trend of number of patients who obtained non-communicable disease management services (hypertension, diabetes mellitus and Asthma) at the study health facilities showed a steady decline in their number in 2020 when compared with the number of cases who obtained the respective services during the same period in the last two years (2018 and 2019) (Fig 2–4).

Compared with 2019 in the same months (March 15 to July 30), the decrease in the number of patients who obtained the respective services in 2020 showed a decrease in 69%, 65% and 57% for hypertension, Diabetes Mellitus and Asthma cases, respectively.

## Discussion

This is the first large scale data to report the impact of the COVID-19 pandemic on the clinical services of non-communicable diseases in Ethiopia. In the present study we found that the health facilities medical services were severely or moderately affected by the COVID-19 pandemic. The under-utilization of important medical services and patients delay despite life threatening symptoms with non-COVID-19 urgent and emergent health needs is an important problem for health care systems 14. Hogan et al showed that, in the era of the COVID-19 pandemic, the lack of proper attention towards non-communicable diseases will lead to greater devastation 15. In the present study, the vast majority of the study participants at the study health centers perceived that the COVID-19 pandemic severely disrupted the non-communicable diseases management services; which is in agreement with a previous finding from Ethiopia 16, which showed that the flow of cases in almost all essential healthcare services were declined as preventive measures against the COVID-19 pandemic.

The decrease in outpatient volume and closure of population level screening programs of non-communicable diseases showed a statistical significant association with the occurrence of the COVID-19 pandemic. This finding showed that the hindrance of the public from regular health service utilization and also outreach community services to find individuals with chronic diseases and conditions but didn't seek health services were aimed at awareness creation as early as possible and making appropriate preventive measures were hampered by the COVID-19 pandemic. A study also indicated that the health status monitoring strategy to identify and solve community health problems have the potential to improve health care delivery adapted to local situation driven by effective health care workers surveillance of households around the world 17.

In a similar fashion, non-communicable disease specific clinical services like the diabetic clinics were closed in some facilities and individuals who were trying to get the consultation and treatment services didn't get access and forced to suffer from the ailments. On the contrary, it is well established that individuals with chronic co-morbidity with diseases and conditions like diabetes were with increased chance of death due to infection with the COVID-19 7,9.

In the current COVID-19 pandemic situation, where accessibility of clinical services is diminished, other healthcare service provision strategies, which seem rewarding in other countries like telehealth service delivery which lacks infrastructure, and inter-practice medical consultation service which is again not well established in Ethiopia need appropriate consideration by responsible institutions in Ethiopia.

Furthermore, managing healthcare crisis associated with shortage of health care professionals and lack of personal protective equipment to provide clinical services for non-communicable diseases in such pandemic situations demand strong collaboration between governmental and non-governmental organizations in Ethiopia.

## Figures and Tables

**Fig 1:**
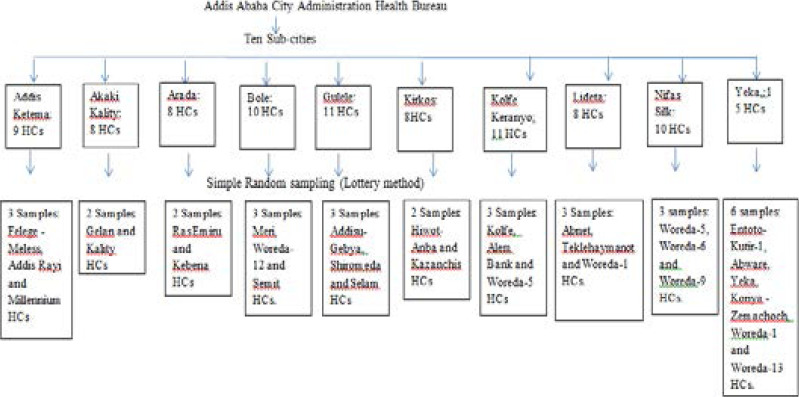
Sampling Frame (HCs: Health centers)

## Conclusion and recommendations

The most critical health-care services and prevention activities for non-communicable diseases were severely disrupted by the COVID-19 pandemic. Responsible institutions striving against non-communicable diseases control including the Ministry of Health of Ethiopia and nongovernmental organizations should provide appropriate attention for the management of non-communicable diseases in the current situation of the COVID-19 pandemic. These responsible bodies should devise a mechanism to ensure availability of clinical services for these diseases regularly, and make the public aware for health service utilization while maintaining universal infection prevention precautions at the health facilities.

## References

[1] World Health Organization Coronavirus disease (COVID-19) outbreak.

[2] World Health Organization (2020). Coronavirus diseases (COVID-19) situation reports.

[3] Reddy K 15 April 2020, Who all are COVID's fellow conspirators?. The New Indian Express.

[4] Gouda H, Charlson F, Sorsdahl K (2019). Burden of non-communicable diseases in sub- Saharan Africa, 1990-2017: results from the Global Burden of Disease Study 2017. Lancet Glob Health.

[5] Institute for Health Metrics and Evaluation Global Health Data Exchange: GBD Results Tool.

[6] N. C. D. Risk Factor Collaboration - Africa Working Group (2017). Trends in obesity and diabetes across Africa from 1980 to 2014: an analysis of pooled population-based studies. Int J Epidemiol.

[7] Roncon L, Zuin M, Rigatelli G, Zuliani G (2020). Diabetic patients with COVID-19 infection are at higher risk of ICU admission and poor short-term outcome. J Clin Virol.

[8] Singh A, Gupta R, Misra A (2020). Comorbidities in COVID-19: Outcomes in hypertensive cohort and controversies with renin angiotensin system blockers. Diabetes Metab Syndr.

[9] Wang B, Li R, Lu Z, Huang Y (2020). Does comorbidity increase the risk of patients with COVID- 19: evidence from meta-analysis. Aging (Albany NY).

[10] WHO (2020). First case of COVID-19 confirmed in Ethiopia.

[11] Ethiopian government COVID-19 Update.

[12] Donabedian A (1980). Methods for deriving criteria for assessing the quality of medical care. Medical Care Review.

[13] Gathogo J (2014). A model for post-implementation valuation of health information systems: the case of the DHIS 2 in Kenya.

[14] Guo H, Zhou Y, Liu X, Tan J (2020). The impact of the COVID-19 epidemic on the utilization of emergency dental services. J Dent Sci.

[15] Hogan A, Jewell B, Sherrard-Smith E, Vesga J, Watson O, Whittaker C (2020). Potential impact of the COVID-19 pandemic on HIV, tuberculosis, and malaria in low-income and middle-income countries: A modelling study. Lancet Glob Health.

[16] (2020). Dessie study: Abdela S, Berhanu A, Ferede L, Griensven J. Essential Healthcare Services in the Face of COVID-19 Prevention: Experiences from a Referral Hospital in Ethiopia. Am. J. Trop. Med. Hyg.

[17] Tulenko K, Vervoort D (2020). Cracks in the System: The Effects of the Coronavirus Pandemic on Public Health Systems. The American Review of Public Administration.

